# Abnormal Lipoproteins Trigger Oxidative Stress-Mediated Apoptosis of Renal Cells in LCAT Deficiency

**DOI:** 10.3390/antiox12081498

**Published:** 2023-07-27

**Authors:** Monica Gomaraschi, Marta Turri, Arianna Strazzella, Marie Lhomme, Chiara Pavanello, Wilfried Le Goff, Anatol Kontush, Laura Calabresi, Alice Ossoli

**Affiliations:** 1 Center E. Grossi Paoletti, Department of Pharmacological and Biomolecular Sciences “Rodolfo Paoletti”, Università degli Studi di Milano, Via Balzaretti 9, 20133 Milan, Italy; monica.gomaraschi@unimi.it (M.G.); marta.turri@unimi.it (M.T.); chiara.pavanello@unimi.it (C.P.); alice.ossoli@unimi.it (A.O.); 2 Foundation for Innovation in Cardiometabolism and Nutrition (ANR-10-IAHU-05), IHU ICAN (ICAN OMICS and ICAN I/O), 75013 Paris, France; m.lhomme@ihuican.org; 3 National Institute for Health and Medical Research (INSERM), UMRS 1166 ICAN, Faculty of Medicine Pitié-Salpêtrière, Sorbonne University, 75013 Paris, France; wilfried.le_goff@upmc.fr (W.L.G.); anatol.kontush@upmc.fr (A.K.)

**Keywords:** LCAT deficiency, renal disease, LpX, preβ-HDL, lipoprotein toxicity, nephrotoxicity

## Abstract

Familial lecithin:cholesterol acyltransferase (LCAT) deficiency (FLD) is a rare genetic disease caused by the loss of function mutations in the *LCAT* gene. LCAT deficiency is characterized by an abnormal lipoprotein profile with severe reduction in plasma levels of high-density lipoprotein (HDL) cholesterol and the accumulation of lipoprotein X (LpX). Renal failure is the major cause of morbidity and mortality in FLD patients; the pathogenesis of renal disease is only partly understood, but abnormalities in the lipoprotein profile could play a role in disease onset and progression. Serum and lipoprotein fractions from LCAT deficient carriers and controls were tested for renal toxicity on podocytes and tubular cells, and the underlying mechanisms were investigated at the cellular level. Both LpX and HDL from LCAT-deficient carriers triggered oxidative stress in renal cells, which culminated in cell apoptosis. These effects are partly explained by lipoprotein enrichment in unesterified cholesterol and ceramides, especially in the HDL fraction. Thus, alterations in lipoprotein composition could explain some of the nephrotoxic effects of LCAT deficient lipoproteins on podocytes and tubular cells.

## 1. Introduction

Familial lecithin:cholesterol acyltransferase (LCAT) deficiency (FLD) is a rare and recessive genetic disease caused by mutations in the *LCAT* gene. It is characterized by marked alterations of the lipid/lipoprotein profile, with a reduction of plasma levels of high-density lipoprotein (HDL) cholesterol variably associated with increased low-density lipoprotein (LDL) cholesterol and triglycerides. Since LCAT is responsible for cholesterol esterification in the plasma compartment, which is key for HDL maturation from discoidal to spherical particles, all lipoprotein fractions are enriched in unesterified cholesterol, and HDL particles are mainly small and discoidal, called preβ-HDL [1]. Moreover, FLD is characterized by the presence of lipoprotein X (LpX), an abnormal lipoprotein detected only in some liver diseases [1,2]. LpX is principally composed of phosphatidylcholine (60%) and unesterified cholesterol (30%) with a diameter between 30 and 70 nm. Under electron microscopy, LpX appears as a multilamellar vesicle with multiple layers of phospholipids [3]. 

Renal failure is the major cause of morbidity and mortality in FLD patients. Early in the disease course, FLD patients develop proteinuria and focal segmental glomerulosclerosis (FSGS) [4]. Even if FLD patients usually develop renal failure with symptomatic edema and hypertension during the third and fourth decade of life [5], the rate of disease progression is unpredictable, with some FLD carriers moving from mild proteinuria to a rapid deterioration of renal function. Kidney histology typically shows FSGS with mesangial expansion, a mild increase in mesangial cellularity, and irregular thickening of the glomerular capillary walls [1]. Lipid deposits with resulting vacuolization of the glomerular basement membrane and a typical “foamy” appearance are also detectable; the lipid analysis of isolated glomeruli shows that the amount of unesterified cholesterol and phospholipids is markedly higher than normal [1]. Besides glomerulopathy, the presence of osmiophilic structures in the basal tubular membrane and in the tubular lumen indicates a tubular alteration in FLD [6]. 

The dramatic changes in lipoprotein profile are likely related to renal disease. In an animal model of FLD, we previously showed that LpX is directly involved in glomerulosclerosis development [7]. However, the cellular and molecular mechanisms of renal injury were not investigated. Furthermore, the potential nephrotoxic role of discoidal preβ-HDL, which accumulates in FLD patients, has not been established yet. 

In this work, the effect of sera and isolated lipoprotein fractions from FLD carriers on podocyte and tubular cell viability was investigated in vitro. To assess the mechanisms responsible for renal damage at a cellular level, changes in markers of cell oxidative stress, inflammation, and functionality were investigated together with features of lipoprotein composition.

## 2. Methods

### 2.1. Subjects 

Fourteen carriers of *LCAT* gene mutations, including 6 carriers of two mutant *LCAT* alleles (HOZ) and 8 heterozygotes (HEZ), all belonging to an Italian cohort of LCAT-deficient families [8], volunteered for the study. HOZ were carriers of LCAT mutations associated with the FLD phenotype, which is associated with renal disease outcome [1]. Eight controls (CTRL) similar for age and gender distribution were also enrolled. 

Each participant signed an informed consent form, which was approved by the Ethics Committee of Milano Area C (approval number 446-092014), in accordance with the principles of the Declaration of Helsinki. Fasting blood was collected, and plasma was prepared by low speed centrifugation at 4 °C. Aliquots were immediately frozen and stored at −80 °C until assayed.

### 2.2. Biochemical Analysis

Plasma levels of total (TC) and HDL cholesterol (HDL-C), triglyceride (TG), apolipoprotein A-I (apoA-I), apoA-II, and apoB were determined with certified methods using a Roche Integra c311 autoanalyzer (Roche Diagnostics, Basel, Switzerland). LDL-C concentration was calculated by Friedwald’s formula. Plasma levels of phospholipids (PL) and unesterified cholesterol (UC) were measured with colorimetric and enzymatic assays, as previously described [9]. The amount of cholesteryl esters (CE) in isolated lipoproteins was calculated as (TC − UC) × 1.68. 

Plasma preβ-HDL content was assessed after separation by 2D electrophoresis followed by immunodetection against human apoA-I (Calbiochem, #178422 dilution 1:1000), and it was expressed as a percentage of total apoA-I [10]. LpX plasma content was detected by FPLC as described [11].

### 2.3. Lipoprotein Isolation for In Vitro Studies

In order to evaluate the nephrotoxicity of the abnormal lipoproteins typically detected in LCAT deficiency, the 1.019–1063 g/mL fraction, containing LDL and LpX if present (defined LDL+LpX fraction), and the 1.063–1.21 g/mL fraction, containing HDL including pre-β particles (called HDL fraction), were isolated from 3 mL of plasma by a multi-step ultracentrifugation in an OptimaTM L-100 XP centrifuge equipped with a 100Ti rotor (Beckman Coulter, Brea, CA, USA) [12]. Plasma density (d = 1.006 g/mL) was adjusted to d = 1.019 g/mL by KBr addition and run at 100,000 rpm for 2 h and 15 min. Then, supernatant was removed, and density was adjusted to d = 1.063 g/mL and run at 100,000 rpm for 2 h and 30 min to isolate LDL. In homozygous LCAT-deficient carriers, LpX floats with LDL due to the same density. The LDL+LpX fraction was collected in the supernatant, while the bottom was adjusted to d = 1.21 g/mL for HDL isolation at 100,000 rpm for 6 h. Both LDL+LpX and HDL fractions were refloated to favor the dissociation of plasma proteins. The isolated lipoprotein fractions were subsequently dialyzed against saline. Lipid and protein composition was evaluated with enzymatic techniques and expressed as a percentage of total mass [13].

### 2.4. Analysis of Sphingolipids in HDL

Lipids were extracted from isolated HDL according to a modified Bligh and Dyer method: a volume of HDL equivalent to 30 μg of PL plasma was supplemented with internal standards, and lipids were extracted with 2.1 mL of CH_3_OH/CHCl_3_ (2:1 *v*/*v*) in the presence of the antioxidant butylated hydroxytoluene (BHT). Then, a total of 550 µL of HCl 0.005N was subsequently added. Phase separation was triggered by the addition of 700 µL of CHCl_3_ and 700 µL of H_2_O and the extracted lipids were dried and re-suspended in 50 µL of LC/MS solvent. Lipids were quantified by LC-ESI/MS/MS using a Prominence UFLC (Shimadzu, Tokyo, Japan) QTrap4000 mass spectrometer (AB Sciex, Framingham, MA, USA). 4 µL of each sample was injected into a Kinetex HILIC 2.6 µm 2.1 × 150 mm column (Phenomenex, Torrance, CA, USA). Mobile phases consisted of (A) H_2_O containing 30 mM of ammonium acetate and 0.2% acetic acid and (B) acetonitrile with 0.2% acetic acid. Lipid species were detected using scheduled multiple reaction monitoring (sMRM) in the positive-ion mode, reflecting the head group fragmentation of each lipid class (Supplemental Table S1). An in-house developed R-script was used to correct for isotopic contribution to MRM signals from HILIC injections based on the approach of Ejsing et al. [14] using the open access R script (enviPat, GPLv2, Martin Loos, Christian Gerber). Raw data were expressed as the mol percentage of total analyzed lipids and recalculated as the mol/g of total protein in HDL and as the circulating plasmatic concentration [15,16]. 

### 2.5. Cell Cultures

In vitro experiments were performed on two different renal cell lines: immortalized human podocytes [17] and human tubular cells (HK2) (CRL-2190, ATCC, Manassas, VA, USA). Podocytes were cultured in Dulbecco′s Modified Eagle Medium (DMEM) containing 1 g/L of glucose supplemented with 10% fetal bovine serum (FBS), 1% L-Glutamine, and 1% antibiotics; HK2 was grown in DMEM F-12 supplemented with 10% FBS, 1% L-Glutamine, and 1% antibiotics. For all the experiments, cells were incubated with sera at 2% *v/v* or with lipoproteins at 0.25 mg/mL of cholesterol.

### 2.6. Evaluation of Apoptosis

To evaluate apoptosis, the activity of caspase 3/7 was measured by luminescence using the Caspase-Glo^®^ 3/7 assay (Promega, Madison, WI, USA) according to the manufacturer’s instructions. Renal cells were seeded in a 96-well plate at a density of 20,000 cells/well and incubated for 48 h with sera from carriers of LCAT mutations or controls. After changing the medium, cells were incubated with proprietary reagent containing the DEVD-aminoluciferin substrate and a thermostable luciferase (100 μL/well) for 2 h in the dark. Luminescence was measured with the Synergy H1 multi-mode reader equipped with the Gen5 software, Version 2.01.14 (BioTek, Agilent, Santa Clara, CA, USA). In each well, the intensity of luminescence was directly proportional to the extent of apoptosis, and the results were expressed as the fold of untreated cells (maintained in 2% *v*/*v* of FBS).

### 2.7. Production of Reactive Oxygen Species (ROS)

ROS production was evaluated as previously described [18]. Briefly, cells were seeded in a 24-well plate at a density of 30,000 cells/well and incubated for 30 min with 5 μM of carboxy-H2DCFDA (Molecular Probes, Invitrogen, Thermo Fisher Scientific, Waltham, MA, USA), a carboxyl derivative of 2′,7′-dichlorofluorescein, in HEPES buffer. Then, cells were treated with sera or isolated lipoprotein fractions from carriers of LCAT mutations and controls for 1 h. The oxidation of the probe was detected by monitoring fluorescence at 517–527 nm with the Synergy H1 multi-mode reader and the Gen5 software (BioTek, Agilent, Santa Clara, CA, USA). For each sample, fluorescence was normalized by the protein concentration of the total cell lysate, measured by the micro-bicinchoninic acid assay (Thermo Scientific, Waltham, MA, USA). 

### 2.8. Gene Expression

After 48 h of incubation with sera or isolated lipoprotein fractions from carriers of LCAT mutations and controls, cells were harvested in Trizol Plus reagent (Life Technologies, Carlsbad, CA, USA), and RNA was extracted according to the manufacturer’s instructions. cDNA was prepared by the reverse transcription of 0.8 μg of total RNA using the iScript cDNA Synthesis kit (Bio-Rad Laboratories, Hercules, CA, USA). Amplification was carried out with the iTaq Universal SYBR Green Supermix in a MiniOpticon System (Bio-Rad Laboratories, Hercules, CA, USA). Amplification consisted of an initial denaturation of the samples at 95 °C for 3 min, followed by 40 cycles of denaturation (95 °C for 10 s), annealing, and amplification (60 °C for 25 s). Expression was calculated using the ΔΔCt method and normalized to β-actin as the housekeeping gene. Primers are reported in Supplementary Table S2.

### 2.9. Statistical Analysis

Data are reported as mean ± SD, unless otherwise stated. Group differences were evaluated by two-tailed one-way ANOVA, and comparisons among the groups were evaluated with a post hoc test (Holm-Sidak method); group differences with a *p* value < 0.05 were considered statistically significant. Analyses were carried out using SigmaPlot 12.5 (Systat Software, Inc., San Jose, CA, USA).

For lipidomic data, an unpaired Student’s *t*-test was performed to compare CTRL vs. HEZ and CTRL vs. HOZ. Two-tailed one-way ANOVA was also performed to find features differentiating the three groups. Group differences with a *p* value < 0.05 after the Benjamini−Hochberg FDR correction were considered statistically significant. Statistical analysis was performed using Multi Experiment Viewer (MeV) software version 4.9.

## 3. Results

### 3.1. Clinical and Biochemical Features of LCAT Deficient Carriers and Controls

All homozygous carriers (HOZ) showed corneal opacity, the hallmark of the disease, and normochromic anemia. Five of the six HOZ also showed renal disease at different stages; specifically, three presented end-stage renal disease (ESRD) that required kidney transplantation, and two had proteinuria. One HOZ presented only with microalbuminuria, an early sign of renal impairment. All HOZ subjects were treated with ACE inhibitors, and two of them were also treated with folic acid. None of the HOZ presented with cardiovascular diseases, in agreement with what was previously reported by our group [19], except for one subject showing hypertension, and mesenteric and aortic plaques requiring surgery for coronary stent implantation. 

The lipid and lipoprotein profile of the enrolled subjects is reported in Table 1. As expected, HOZ showed a significant decrease in plasma levels of HDL-C and a marked increase of UC concentration and, consequently, of the UC/TC ratio compared to HEZ and controls, in a gene–dose-dependent fashion. A significant reduction of apoA-I and apoB concentrations in HOZ was evident as well. HOZ also had higher concentrations of triglycerides and phospholipids compared to the other groups. The percentage of preβ-HDL showed a significant gene–dose-dependent increase in carriers of LCAT mutations. The abnormal lipoprotein LpX was detected in all HOZ subjects, while it was not detectable in any HEZ or control subject.

### 3.2. Lipoprotein Composition

Firstly, lipoproteins isolated from HOZ, HEZ, and controls were characterized in terms of mass composition (Table 2). Since LpX, present only in the plasma of homozygous FLD carriers, is isolated from plasma at the same density of LDL, the LDL fraction was identified in the manuscript as the LDL+LpX fraction. Both LDL+LpX and HDL from HOZ showed an altered composition if compared to HEZ and the controls. The LDL+LpX fraction of HOZ was significantly enriched in unesterified cholesterol and phospholipids, likely due to the presence of LpX, as well as in triglycerides; on the contrary, the percentage of cholesteryl esters and protein was markedly reduced compared to LDL+LpX from HEZ and the controls. Similarly, the HDL fraction of HOZ, that contains mainly preβ-HDL particles, showed an increased content of phospholipids and unesterified cholesterol and a reduced protein content compared to HEZ and the controls. As previously reported [20], cholesteryl esters were completely absent in HOZ HDL.

To further characterize HDL lipid composition, a lipidomic analysis was performed to evaluate the content and composition of sphingolipids, minor but biologically active lipid species. Dihydrosphingomyelin (DHSM), sphingomyelin (SM), ceramides (Cer 18:1;O2), sphingadienines (Cer 18:2;O2), and dihydroceramides (DHC) were measured (Table 3). The analysis of sphingolipids highlighted a significant enrichment in DHC and Cer 18:1 expressed as mol% in HOZ HDL compared to control HDL. Moreover, even if the number of circulating HDL was low in HOZ, the absolute concentrations of HDL-associated DHC and Cer in the circulation (as nmol/µL) were not reduced when compared to the controls. HEZ HDL did not show significant changes in sphingolipid classes compared to the controls.

### 3.3. Induction of Renal Cell Apoptosis by Sera from LCAT Deficient Carriers

**Figure 1:**
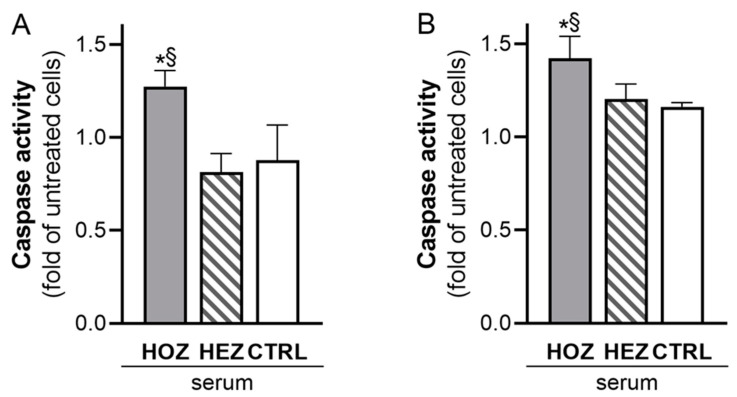
LCAT-deficient serum promotes the apoptosis of podocytes and tubular cells. Caspase 3/7 activity induced by incubation of podocytes (**A**) and tubular cells (**B**) with subjects’ sera. Data are expressed as the fold of untreated cells, mean ± SEM. Data were analyzed by two-tailed one way ANOVA (*p* < 0.001 for podocytes and *p* = 0.005 for tubular cells). All pairwise multiple comparisons were performed by the Holm-Sidak method ^§^ *p* < 0.01 vs. HEZ, * *p* < 0.01 vs. ctrl. HOZ (n = 5), HEZ (n = 5), and CTRL (n = 4). HOZ, homozygous carriers; HEZ, heterozygous carriers; CTRL, controls.

Sera from carriers of LCAT mutations and controls were tested for their effect on renal cell apoptosis (Figure 1). Sera from HOZ significantly induced the apoptosis of podocytes, while those from HEZ and controls did not; indeed, caspase 3/7 activation increased by 56.5 ± 10.6% with HOZ sera compared to HEZ sera (*p* = 0.001) and by 45.1 ± 9.9% compared to control sera (*p* < 0.001, Figure 1A). HOZ sera induced the apoptosis of tubular cells as well (Figure 1B). The incubation of tubular cells with HOZ sera induced a significantly higher caspase 3/7 activation compared to HEZ and control ones (+18.3 ± 9.7% vs. HEZ, *p* = 0.01; +22.4 ± 10.1% vs. controls, *p* = 0.009). Caspase 3/7 data were confirmed by performing annexin V labelling of podocytes incubated for 48 h with sera from carriers and controls. Sera from HOZ almost doubled the percentage of apoptotic cells (positive for annexin V staining), while no difference was observed between sera from HEZ and controls (Supplemental Figure S2).

To confirm the toxicity of plasma lipoproteins on renal cell apoptosis, caspase 3/7 activation was evaluated using isolated LpX and HDL fractions from HOZ carriers and controls. The results clearly showed that both LpX and HDL isolated from HOZ carriers induced higher caspase 3/7 activation than lipoproteins isolated from controls (Supplemental Figure S1).

### 3.4. Induction of ROS by Sera and Lipoproteins from LCAT Deficient Carriers

**Figure 2:**
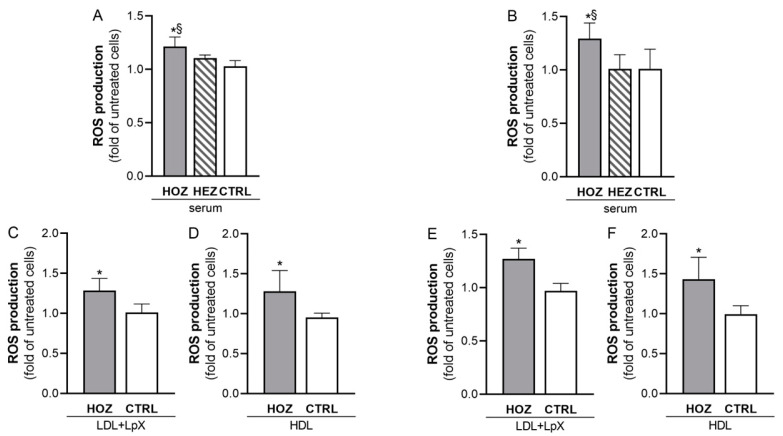
LCAT deficient serum and lipoproteins mediated ROS production in podocytes and in tubular cells. ROS production was induced by the incubation of podocytes (**A**) and tubular cells (**B**) with sera from LCAT-deficient subjects and their controls. Data are expressed as the fold of untreated cells, mean ± SEM, ^§^ two-tailed *p* < 0.01 vs. HEZ, * two-tailed *p* < 0.05 vs. CTRL. HOZ (n = 4), HEZ (n = 4), and CTRL (n = 8). (**C**–**F**): ROS production in podocytes (**C**,**D**) and in tubular cells (**E**,**F**) incubated with LDL+LpX (**C**,**E**) and HDL (**D**,**F**) isolated from homozygotes’ and controls’ sera. Data are expressed as the fold of untreated cells, mean ± SEM * two tailed *p* < 0.05. HOZ (n = 4), CTRL (n = 4). HOZ (n = 5), HEZ (n = 5), and CTRL (n = 4). HOZ, homozygous carriers; HEZ, heterozygous carriers; CTRL, controls.

To understand the molecular mechanisms behind the induction of renal cell apoptosis by HOZ sera, the possible involvement of oxidative stress was investigated since it has been previously shown to impair both glomerular and tubular functions [21]. To this aim, the ability of LCAT-deficient sera to induce oxidative stress in immortalized human podocytes and tubular cells was tested (Figure 2A,B). Only HOZ sera triggered a significant increase of ROS production in both podocytes and HK2. Compared to control sera, HOZ sera increased ROS levels in podocytes by 18.2 ± 8.6%, (*p* < 0.001) and in HK2 by 27.7 ± 14.6% (*p* = 0.035), thus supporting the induction of oxidative damage both at glomerular and at tubular levels. HEZ sera did not affect ROS production in both cell lines.

To assess whether the increase of oxidative stress was mediated by the accumulation of LpX and preβ-HDL in HOZ sera, renal cells were incubated with LDL+LpX and HDL fractions isolated from HOZ and controls, and the consequent ROS production was measured (Figure 2C–F). In podocytes, both LDL+LpX and HDL fractions from HOZ significantly increased ROS levels compared to lipoproteins isolated from controls (+26.9 ± 14.8% with LDL+LpX fraction, *p* = 0.025; +34.0 ± 27.3% with HDL, *p* = 0.050) (Figure 2C,D). LpX and preβ-HDL are also involved in the increased oxidative stress of tubular cells; indeed, ROS production increased by 25.4 ± 10.0% with the LDL+LpX fraction of HOZ when compared to control ones (*p* = 0.013) and by 50 ± 28.7% with HOZ HDL when compared to control ones (*p* = 0.025) (Figure 2E,F). Thus, LpX and preβ-HDL play a direct role in inducing the oxidative damage of renal cells in FLD.

### 3.5. Lipoprotein-Independent Induction of Inflammation by Sera from LCAT Deficient Carriers

**Figure 3:**
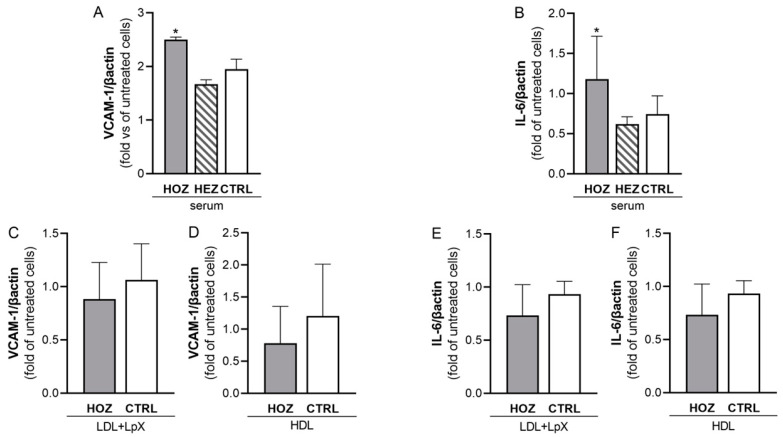
Inflammation induced by LCAT-deficient serum and lipoproteins in tubular cells. Interleukin-6 (**A**) and VCAM-1 (**B**) expression induced by the incubation of serum in tubular cells. Data were analyzed by two-tailed one way ANOVA. * *p* < 0.05 vs. ctrl. HOZ (n = 4), HEZ (n = 4), and CTRL (n = 4). IL-6 expression mediated by LDL+LpX (**C**) and HDL (**D**) isolated from homozygous’ and controls’ plasma. VCAM-1 expression mediated by LDL+LpX (**E**) and HDL (**F**) isolated from homozygous’ and controls’ plasma. Data are expressed as mean ± SEM. HOZ (n = 3), CTRL (n = 3). HOZ, homozygous carriers; HEZ, heterozygous carriers; CTRL, controls.

Kidney tubular epithelial cells operate as professional immune cells by releasing several cytokines, as interleukin-6 (IL-6), and by directly interacting with neutrophils, monocytes, and T cells through the expression of cell adhesion molecules, such as vascular cell adhesion molecule 1 (VCAM-1) [22]. Thus, the effect of sera and lipoprotein fractions on IL-6 and VCAM-1 expression by HK2 was tested as well. When HK2 was incubated with HOZ sera, an increased expression of both IL-6 and VCAM-1 was observed (Figure 3A,B). However, this effect was not mediated by abnormal lipoproteins in HOZ sera, since IL-6 and VCAM-1 mRNA levels were comparable in HK2 incubated with LDL+LpX and HDL fractions from HOZ and controls (Figure 3C–F).

### 3.6. Inhibition of Podocin and Synaptopodin Expression by Sera and Lipoproteins from LCAT Deficient Carriers

**Figure 4:**
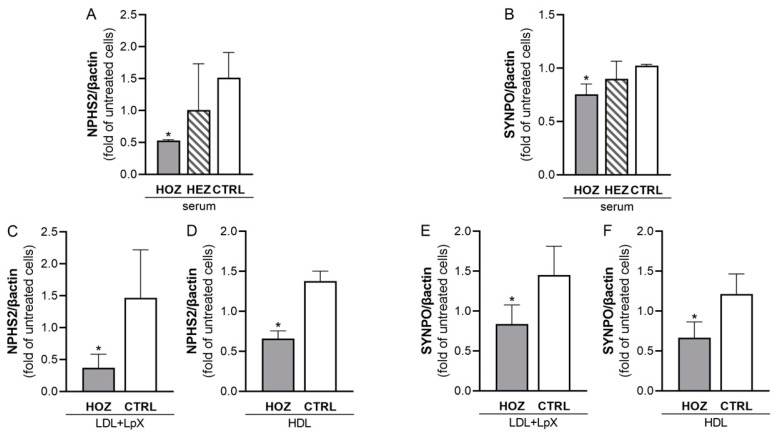
LCAT-deficient serum and lipoprotein-mediated podocin and synaptopodin gene expression. The expression of genes coding for podocin (NPHS2, (**A**)) and synaptopodin (SYNPO, (**B**)) induced by serum in podocytes was quantified by qPCR. Data were analyzed by two-tailed one way ANOVA. Data are expressed as the fold of untreated cells, mean ± SEM, * *p* < 0.05 vs. CTRL. HOZ (n = 4), HEZ (n = 4), and CTRL (n = 4). (**C**,**D**): NPHS2 expression mediated by LDL+LpX and HDL isolated from homozygous’ and controls’ plasma. (**E**,**F**): SYNPO expression mediated by LDL+LpX and HDL isolated from homozygous’ and controls’ plasma. Data are expressed as mean ± SEM, * *p* < 0.05. HOZ (n = 4), CTRL (n = 4). HOZ, homozygous carriers; HEZ, heterozygous carriers; CTRL, controls.

In podocytes, podocin and synaptopodin are instrumental in the correct slit-diaphragm function during ultrafiltration; thus, their gene expression after incubation with sera from LCAT-deficient carriers and control subjects was assessed [23]. Sera from FLD patients induced a significant reduction of the expression of both *NPHS2* (coding for podocin) and *SYNPO* genes (coding for synaptopodin); indeed, HOZ sera significantly inhibited *NPHS2* expression by 65.2 ± 1.1% and *SYNPO* expression by 26.2 ± 9.5% when compared to control ones (*p* < 0.001 and *p* = 0.007, respectively) (Figure 4A,B). To assess whether the inhibition of *NPHS2* and *SYNPO* expression affecting podocyte functionality was mediated by the accumulation of LpX and preβ-HDL in HOZ sera, renal cells were incubated with LDL+LpX and HDL fractions isolated from HOZ and controls (Figure 4C–F). The LDL+LpX fraction of HOZ reduced *NPHS2* expression by 69.9 ± 17.9% and *SYNPO* by 29.8 ± 20.1%, if compared to the control LDL+LpX fraction, in which LpX was not present (*p* = 0.030 and *p* = 0.029, respectively). Similarly, HOZ HDL reduced *NPHS2* expression by 50.3 ± 7.2% and *SYNPO* by 49.7 ± 14.8% if compared to control HDL (*p* < 0.001 and *p* = 0.014, respectively). These data further confirm the direct involvement of LpX and preβ-HDL in the impairment of glomerular function.

## 4. Discussion

The present work showed, for the first time, that (i) the serum of homozygous FLD carriers is damaging for podocytes and tubular cells, (ii) the abnormal LpX and HDL particles that specifically accumulate in the plasma of homozygous FLD carriers are direct mediators of the nephrotoxic effect, and (iii) critical mechanisms of lipoprotein nephrotoxicity are ROS production and apoptosis induction.

Familial LCAT deficiency is a very rare disorder of lipid metabolism with important clinical consequences [24] and presently no cure. FLD is typically associated with lipoprotein abnormalities, which are evident in homozygous but not in heterozygous carriers [24]. HDL are dramatically reduced, and circulating particles are all discoidal and immature with virtually no cholesteryl esters, underlying the key role of LCAT in HDL maturation. In addition, LpX accumulates in plasma to accommodate the large amount of circulating unesterified cholesterol and phospholipids. The analysis of lipoprotein fractions isolated in the present study confirmed the dramatic alterations observed in all lipoproteins from homozygous FLD carriers. Early studies from our group have already shown that LpX is at least partly responsible for the renal damage associated with LCAT deficiency [7], and here, we showed that FLD HDL particles can also contribute to renal damage. Both these lipoprotein fractions are particularly enriched in unesterified cholesterol, which deposits in glomeruli, as shown by the lipid analysis of kidney biopsies of LCAT-deficient patients [2]. The key role of unesterified cholesterol in kidney damage is further supported by the observation that the plasma unesterified cholesterol level has been identified as an independent predictor of the rapid deterioration of kidney function in FLD patients [5]. Unesterified cholesterol is known to directly induce cell toxicity by several mechanisms, including the disruption of membrane domains, the induction of apoptosis, and the formation of toxic oxidized molecules [25]. 

In the present study, we showed that the LpX and HDL of homozygous FLD carriers induce oxidative damage in tubular and glomerular cells. ROS production has been associated with podocyte damage in several experimental models of renal disease, including diabetic nephropathy, membranous nephropathy, minimal change disease, and FSGS [26]. Oxidative stress can also directly affect tubular cells, as shown in acute kidney injury and in tubule-interstitial fibrosis [21,27]; due to their high energy request to support the active reabsorption processes, tubular cells are enriched in mitochondria, a site of strong ROS production. Oxidative stress appears at the early stages of chronic kidney disease (CKD), and it plays a key role in the pathogenesis of renal failure since it impairs both glomerular and tubular functions by inducing cell apoptosis; indeed, ROS was shown to trigger an intricate cascade of events leading to caspase activation [21,26,28]. HDL is typically considered to have antioxidant activity [29]; however, the abnormal enrichment in unesterified cholesterol of FLD HDL likely explains their pro-oxidant activity; in fact, it has been shown that HDL’s ability to inhibit LDL oxidation is inversely associated with HDL unesterified cholesterol content [29]. In addition, HDL from homozygous FLD carriers are enriched in pro-oxidant ceramides and DHC, a well-known pro-apoptotic factor, as previously shown [30,31] and confirmed by our lipidomic analysis. 

Under physiological conditions, aerobic cells are endowed with extensive antioxidant mechanisms to counteract the damaging effects of ROS [32]. However, in pathological conditions, excessive oxidative stress can trigger cell necrosis or apoptosis, with the latter also acting as a fail-safe device to prevent amok proliferation [33]. Here, we showed that sera from homozygous FLD carriers induce renal cell apoptosis. This is in line with what was previously observed in primary and secondary forms of FSGS, where the depletion of podocytes represents the central manifestation of chronic progressive glomerular diseases. Indeed, subjects affected by glomerulopathy usually present with a reduced expression of podocin and synaptopodin, key molecules for the maintenance of slit-diaphragm function, which is the main unit of the ultrafiltration barrier [23]. Consistently, mutations in the genes coding for these proteins have been identified in several families with autosomal recessive FSGS, with recessive familial nephrotic syndromes, and in HIV-associated nephropathy [34,35]. The reduced expression of podocin and synaptopodin, two crucial proteins for podocyte function, in cells exposed to LpX and HDL fractions from homozygous FLD carriers suggests that FLD lipoproteins could directly impair the ultrafiltration process. 

In conclusion, collected data indicate that the abnormal lipoproteins that accumulate in the plasma of FLD patients are likely directly responsible for kidney damage, leading to the worst clinical complication of the disease. The results suggest that the normalization of plasma lipoproteins, by promoting LpX degradation and HDL maturation, is mandatory to treat this rare disease presently with no cure. Indeed, FLD patients are currently managed with pharmacological approaches aimed at correcting the dyslipidemia typically associated with the disease and at delaying the evolution of chronic nephropathy. FLD patients are also candidates for kidney transplantation, but the pathology reoccurs within a few years [5], confirming that the systemic alterations must be corrected to cure FLD. We have recently shown that treatment with an HDL mimetic can remodel plasma lipoproteins, thus reducing podocyte lipid deposit [36,37]. Other potential pharmacological approaches include the use of small molecules activating LCAT [38,39,40] and of recombinant human LCAT, for which development has been discontinued [41]. In the meanwhile, the identification of the nephrotoxic pathways involved in the onset of renal disease in FLD carriers may improve current strategies to delay renal impairment.

In interpreting these results, the following limitations should be taken into account: (i) the results were obtained with a small number of samples; LCAT deficiency is an ultra-rare disease, and despite that we have collected a large cohort of carriers, the availability of plasma volumes necessary to isolate lipoproteins and run the in vitro experiments was limited, and (ii) the study conclusions are based on ex vivo and in vitro findings and further studies are warranted to demonstrate their translation in vivo.

## Figures and Tables

**Table 1:** Characteristics of the subjects.

	HOZ	HEZ	CTRL	*p*-Value
N	6	8	8	
Sex (m/f)	5/1	5/3	4/4	0.201
Age	37.8 ± 9.3	57.0 ± 15.1	36.3 ± 7.7	0.111
Total cholesterol (mg/dL)	154.3 ± 71.0	185.9 ± 48.7	194 ± 20.3	0.663
Unesterified cholesterol (mg/dL)	122.0 ± 58.1 ^§^*	53.5 ± 14.9	26.4 ± 16.9	0.002
UC/TC (%)	79.2 ± 15.3 ^§^*	29.7 ± 8.0	14.3 ± 9.9	<0.001
LDL-cholesterol (mg/dL)	83.2 ± 43.7.0	119.0 ± 45.7	115.7 ± 26.4	0.228
HDL-cholesterol (mg/dL)	7.0 ± 1.4 ^§^*	38.9 ± 9.9	64.7 ± 20.8	<0.001
Triglycerides (mg/dL)	321.0 ± 266.9 ^§^*	139.6 ± 51.8 *	73.6 ± 27.0	0.007
Phospholipids (mg/dL)	330.4 ± 88.3 ^§^*	226.4 ± 39.5	239.7 ± 42.7	0.017
apoA-I (mg/dL)	37.0 ± 10.8 ^§^*	116.1 ± 24.3	120.8 ± 25.2	<0.001
apoA-II (mg/dL)	4.9 ± 1.3 ^§^*	32.6 ± 3.8	29.5 ± 2.1	<0.001
apoB (mg/dL)	62.3 ± 33.1 ^§^*	107.3 ± 29.9	93.5 ± 15.1	0.035
Preβ-HDL (%)	51.0 ± 11.5 ^§^*	23.9 ± 3.2	12.1 ± 1.1	<0.001
LpX (+/−)	6/0 ^§^*	0/8	0/8	<0.001

Data are expressed as mean ± SD except for categorical variables. Continuous variables were analyzed by two-tailed one way ANOVA. All pairwise multiple comparisons were performed by the Holm-Sidak method. Categorical variables were analyzed by a Chi-Square test. ^§^ *p* < 0.05 vs. HEZ, * *p* < 0.05 vs. CTRL. TC, total cholesterol; UC, unesterified cholesterol; apoA-I, apolipoprotein A-I; apoA-II, apolipoprotein A-II; apoB, apolipoprotein B; LpX, lipoprotein X; HOZ, homozygous carriers; HEZ, heterozygous carriers; CTRL, controls.

**Table 2:** Composition of isolated lipoprotein fractions.

LDL+LpX				
	HOZ	HEZ	CTRL	*p*-Value
UC (%)	19.6 ± 3.9 ^§^*	9.0 ± 1.6	9.0 ± 2.3	<0.001
CE (%)	4.1 ± 4.2 ^§^*	30.3 ± 10.0	37.1 ± 5.7	<0.001
PL (%)	42.0 ± 6.4 ^§^*	25.4 ± 4.4	22.5 ± 5.7	<0.001
TG (%)	23.0 ± 5.1 ^§^*	14.6 ± 3.3 *	9.0 ± 2.3	0.016
PT (%)	11.3 ± 4.8 ^§^*	21.0 ± 4.4	22.2 ± 6.0	<0.001
**HDL**				
	**HOZ**	**HEZ**	**CTRL**	***p*-Value**
UC (%)	17.7 ± 2.8 ^§^*	3.9 ± 1.2	3.8 ± 0.9	<0.001
CE (%)	0.3 ± 0.8 ^§^*	16.0 ± 3.1 *	21.6 ± 3.9	<0.001
PL (%)	45.9 ± 3.1 ^§^*	29.4 ± 5.9	32.0 ± 5.6	<0.001
TG (%)	4.9 ± 2.2	5.6 ± 1.1	3.6 ± 1.1	0.011
PT (%)	31.3 ± 3.5 ^§^*	45.4 ± 7.6	39.3 ± 7.3	0.003

Data are expressed as the percentage of total mass, mean ± SD. Parameters were analyzed by two-tailed one way ANOVA. All pairwise multiple comparisons were performed by the Holm-Sidak method. ^§^ *p* < 0.05 vs. HEZ, * *p* < 0.05 vs. ctrl. HOZ (*n* = 6), HEZ (*n* = 8), CTRL (*n* = 8). UC, unesterified cholesterol; CE, esterified cholesterol; TG, triglycerides; PL, phospholipids; PT, protein.

**Table 3:** HDL lipidome.

	Fold Change HEZ vs. CTRL			Fold Change HOZ vs. CTRL		
	Mol % (PL + SL)	mol/g prot.	nmol/μL	Mol % (PL + SL)	mol/g prot.	nmol/μL
Total SM	0.98	0.92	0.81	1.01	1.25	0.54
Total DHSM	0.86	0.81	0.73	1.21	1.44	0.63
Total DHC	1.50	1.40	1.23	4.96 *	6.09 *	2.71
Total Cer 18:1;O2	1.23	1.14	1.00	2.33 *	2.78	1.21
Total Cer 18:2;O2	0.96	0.90	0.79	1.26	1.61	0.69

Table shows the fold change values of HEZ or HOZ vs. controls calculated from sphingolipids expressed as the mol% of total phospholipids + sphingolipids, mol/g of total protein, or nmol/μL in plasma. * two-tailed *p* < 0.05 vs. controls (CTRL). Phospholipids (PL), sphingolipids (SL), sphingomyelin (SM), dihydrosphingomyelin (DHSM), ceramide (Cer d18:1;O2), sphingadienines (Cer 18:2;O2), dihydroceramide (DHC). HOZ (*n* = 6), HEZ (*n* = 8), CTRL (*n* = 8).

## Data Availability

Data are available upon reasonable request to the corresponding authors.

## Supplementary Materials

The following supporting information can be downloaded at: https://www.mdpi.com/article/10.3390/antiox12081498/s1. Supplementary Table S1. Conditions of mass spectrometry analysis; Supplementary Table S2. Primers for real-time PCR; Supplementary Figure S1. Effect of isolated lipoproteins on apoptosis of renal cells; Supplementary Figure S2. Evaluation of apoptosis of podocytes by annexin V staining.
