# Supplementary material for: Abnormal Lipoproteins Trigger Oxidative Stress-Mediated Apoptosis of Renal Cells in LCAT Deficiency
Source: Antioxidants (Basel). 2023 Jul 27;12(8):1498. doi: 10.3390/antiox12081498 (PMC10451761; doi:10.3390/antiox12081498)
Supplement: Supplementary file 1 [file antioxidants-12-01498-s001.zip › antioxidants-2479944-supplementary.pdf]

SUPPLEMENTAL MATERIAL

Abnormal Lipoproteins Trigger Oxidative Stress-Mediated Apoptosis of Renal Cells in LCAT Deficiency

Monica Gomaraschi [1], Marta Turri [1], Arianna Strazzella [1], Marie Lhomme [2], Chiara Pavanello [1], Wilfried Le Goff [3], Anatol Kontush [3], Laura Calabresi [1,*] and Alice Ossoli [1]

[1] Center E. Grossi Paoletti, Department of Pharmacological and Biomolecular Sciences "Rodolfo Paoletti", Università degli Studi di Milano, Via Balzaretti 9, 20133 Milan, Italy

[2] Foundation for Innovation in Cardiometabolism and Nutrition (ANR-10-IAHU-05), IHU ICAN (ICAN OMICS and ICAN I/O), 75013 Paris, France

[3] National Institute for Health and Medical Research (INSERM), UMRS 1166 ICAN, Faculty of Medicine Pitié-Salpêtrière, Sorbonne University, 75013 Paris, France

Supplementary Table S1. Conditions of mass spectrometry analysis

<table>
<tr><th>Sphingolipids' subclasses</th><th>N of assayed species</th><th>N of quantified species (>LOQ)</th><th>ISTD</th><th>Parent ion</th><th>MS Experiment</th></tr>
<tr><td>Sphingomyelin (SM)</td><td>16</td><td>16</td><td rowspan="2">SM (d18:1/18:1-d9)</td><td rowspan="2">[M+H]+</td><td rowspan="2">PIS 184m/z</td></tr>
<tr><td>Dihydrosphingomyelin (DHSM)</td><td>9</td><td>8</td></tr>
<tr><td>Ceramide (Cer d18:1;O2)</td><td>14</td><td>13</td><td rowspan="3">Cer (d18:1-d7/24:0)</td><td rowspan="2">[M+H]+</td><td>PIS 264m/z</td></tr>
<tr><td>Sphingadienines (Cer 18:2;O2)</td><td>11</td><td>10</td><td>PIS 262m/z</td></tr>
<tr><td>Dihydroceramide (Cer 18:0;O2 DHC)</td><td>11</td><td>10</td><td>[M+H]+</td><td>PIS 266m/z</td></tr>
</table>

LOQ: limit of quantitation, ISTD: internal standard, PIS: product Ion Scan

Supplementary Table S2. Primers for real-time PCR

Target	*Primer forward*	*Primer reverse*
β-actin	CTG GAC TTC GAG CAA GAG ATG	CCA TGC CCA GGA AGG AAG
Podocin	CAT GAG ATC GTG ACC AAA GAC	GAG ACG CTT CAT AGT GGT TTG
Synaptopodin	AAG TCA CAT CCA GCT CCT TC	CTT CTC CGT GAG GCT AGT G
IL-6	AAC CTG AAC CTT CCA AAG ATG G	TCT GGC TTG TTC CTC ACT ACT
VCAM-1	CCA CAG TAA GGC AGG CTG TAA AAG	CGC TGG AAC AGG TCA TGG TCA C

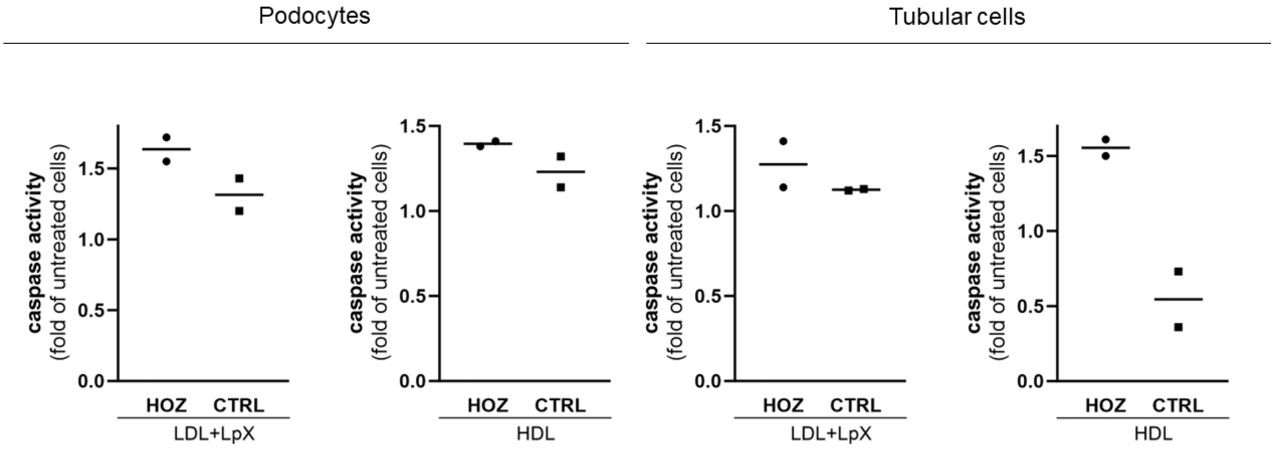

Supplementary Figure S1. Effect of isolated lipoproteins on apoptosis of renal cells. Caspase 3/7 activity induced by incubation of podocytes and tubular cells with LDL+LpX or HDL isolated from HOZ (n=2) and CTRL (n=2). HOZ, homozygous carriers; CTRL, controls.

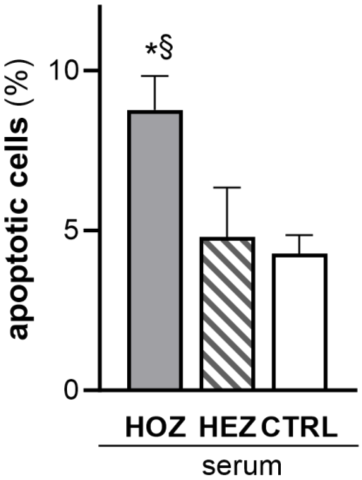

Supplementary Figure S2. Evaluation of apoptosis of podocytes by annexin V staining. The percentage of apoptotic cells (positive for annexin V staining) on total cells is reported as mean±SEM. Data were analyzed by One Way ANOVA $P=0.005$, § P(two-tailed) <0.05 vs HEZ, *P(two-tailed) <0.05 vs ctrl. HOZ (N=3), HEZ (N=3) and CTRL (N=3). HOZ, homozygous carriers; HEZ, heterozygous carriers; CTRL, controls.
